# Exposure to pairs of *Aeromonas* strains enhances virulence in the *Caenorhabditis elegans* infection model

**DOI:** 10.3389/fmicb.2015.01218

**Published:** 2015-11-04

**Authors:** Thomas Mosser, Emilie Talagrand-Reboul, Sophie M. Colston, Joerg Graf, Maria J. Figueras, Estelle Jumas-Bilak, Brigitte Lamy

**Affiliations:** ^1^Laboratoire de Bactériologie-Virologie, Équipe Pathogènes Hydriques Santé Environnements, UMR 5569 HydroSciences Montpellier, Université de MontpellierMontpellier, France; ^2^Département d'Hygiène Hospitalière, Centre Hospitalier Régional Universitaire de MontpellierMontpellier, France; ^3^Department of Molecular and Cell Biology, University of ConnecticutStorrs, CT, USA; ^4^Institute for Systems Genomics, University of ConnecticutStorrs, CT, USA; ^5^Unidad de Microbiología, Facultad de Medicina y Ciencias de la Salud, Universidad Rovira i VirgiliReus, Spain; ^6^Laboratoire de Bactériologie, Centre Hospitalier Régional Universitaire de MontpellierMontpellier, France

**Keywords:** mixed infection, polymicrobial infection, *Aeromonas*, *Caenorhabditis elegans*, virulence determinants

## Abstract

Aeromonad virulence remains poorly understood, and is difficult to predict from strain characteristics. In addition, infections are often polymicrobial (i.e., are mixed infections), and 5–10% of such infections include two distinct aeromonads, which has an unknown impact on virulence. In this work, we studied the virulence of aeromonads recovered from human mixed infections. We tested them individually and in association with other strains with the aim of improving our understanding of aeromonosis. Twelve strains that were recovered in pairs from six mixed infections were tested in a virulence model of the worm *Caenorhabditis elegans*. Nine isolates were weak worm killers (median time to death, TD_50_, ≥7 days) when administered alone. Two pairs showed enhanced virulence, as indicated by a significantly shortened TD_50_ after co-infection vs. infection with a single strain. Enhanced virulence was also observed for five of the 14 additional experimental pairs, and each of these pairs included one strain from a natural synergistic pair. These experiments indicated that synergistic effects were frequent and were limited to pairs that were composed of strains belonging to different species. The genome content of virulence-associated genes failed to explain virulence synergy, although some virulence-associated genes that were present in some strains were absent from their companion strain (e.g., T3SS). The synergy observed in virulence when two *Aeromonas* isolates were co-infected stresses the idea that consideration should be given to the fact that infection does not depend only on single strain virulence but is instead the result of a more complex interaction between the microbes involved, the host and the environment. These results are of interest for other diseases in which mixed infections are likely and in particular for water-borne diseases (e.g., legionellosis, vibriosis), in which pathogens may display enhanced virulence in the presence of the right partner. This study contributes to the current shift in infectiology paradigms from a premise that assumes a monomicrobial origin for infection to one more in line with the current pathobiome era.

## Introduction

Aeromonads are ubiquitous free-living organisms that are found mainly in aquatic environments. These bacteria are opportunistic pathogens that are involved in various types of infections in a wide range of hosts, including fish and humans. Human infections occur as a consequence of either traumatic occupational injuries or exposure during recreational activities, leech therapy, or water or food consumption (Wiklund and Dalsgaard, 1998; Janda and Abbott, 2010; Figueras and Beaz-Hidalgo, 2015). The outcomes of *Aeromonas* infections vary from mild to life threatening, including in immunocompetent patients (Janda and Abbott, 2010; Shak et al., 2011; Figueras and Beaz-Hidalgo, 2015). The virulence potential of any given strain remains difficult to predict (Albert et al., 2000; Martins et al., 2002; Chacón et al., 2003; Senderovich et al., 2012), and it has been suggested that only certain subsets of strains can produce disease in certain individuals (Janda and Abbott, 1998, 2010; Joseph and Carnahan, 2000). These specific subsets of strains have been referred to as pathotypes in more recent studies (Grim et al., 2013). However, population studies aimed at discerning differences in the repertoires of virulence factors between strains recovered from clinical samples and environmental sources found either that there was no difference between these two isolation sources or contradictory results (Albert et al., 2000; Martins et al., 2002; Chacón et al., 2003; Vilches et al., 2009; Senderovich et al., 2012). Generally, aeromonads carry in their genomes numerous virulence factor genes, which has led to their “Jack-of-all-Trades” status (Seshadri et al., 2006), but the presence of a gene does not necessarily equate to its expression, which may depend on the context in the host or niche (Vilches et al., 2009).

Infections caused by *Aeromonas* (hereafter aeromonosis) often involve more than one type of bacteria within the same clinical sample (so-called polymicrobial or mixed infections). Mixed infections occur with a frequency that ranges from 30% for bacteremia to 60% for wound infection and even 80% in the case of respiratory tract infections (Lamy et al., 2009; Figueras and Beaz-Hidalgo, 2015). Aeromonads are mainly associated with enterobacteria, *Staphylococcus aureus*, or anaerobes (Lamy et al., 2009), but there is an interesting subgroup of mixed infection cases that involves the presence of two distinct *Aeromonas* strains in the same sample. The incidence of this phenomenon is surprising and frequent enough to regularly attract the attention of scientists (Joseph et al., 1979, 1991; Shak et al., 2011). From a previous study, we estimated its minimum frequency as 5% of aeromonosis (Lamy et al., 2009).

Increasing evidence suggests that complex relationships exist between bacteria from distinct species or genera in the setting of an infection (Crane et al., 2006; Lo et al., 2011; Ramsey et al., 2011; Peters et al., 2012; Korgaonkar et al., 2013). However, little is known regarding the implications of the poly-aeromonad samples. In an attempt to improve understanding of these mixed infections from an ecological and pathogenic point of view, we aimed to evaluate the pathogenic behavior of *Aeromonas* strains recovered from mixed infections, both individually and in association with other strains, as a test case of *Caenorhabditis elegans* as a virulence model. We evaluated six aeromonad pairs that co-occurred in the same clinical samples and 14 other experimental pairs. In up to 36% of the pairs we tested, we identified synergistic pathogenic behavior. This finding may contribute to a better understanding of the pathogenicity of aeromonads.

## Methods

### Strain collection and culture conditions

A total of six natural pairs of *Aeromonas* strains (12 strains) belonging to five species were collected from various samples (Table 1). Strains were studied alone or in combination with their natural co-isolate. Three other strains were recovered as single isolates (Table 1). The avirulent *E.coli* strain OP50, which is a standard food for nematodes that does not possess known virulence factors, was also included in the study. All strains were identified at the species level by sequencing the *gyrB* and *rpoB* gene products using PCR, as previously described (Yáñez et al., 2003; Korczak et al., 2004). Bacteria were grown in trypticase soy broth (TSB) or agar (TSA) at 37°C.

**Table 1:** **Origin of the strains used in this study**.

**Species**	**Strain**	**Clinical sample pair**	**Origin**	**Region, country, and year of isolation**	**WGS reference**
*A. media*	76c	1	Stool	Barcelona, Spain, 1992	PRJEB8966
*A. veronii*	77c	1	Stool	Barcelona, Spain, 1992	PRJEB9012
*A. caviae*	388c	2	Stool	Barcelona, Spain, 2000	–
*A. caviae*	404c	2	Stool	Barcelona, Spain, 2000	–
*A. hydrophila*	BVH 25a	3	Respiratory tract	Saint-Brieux, France, 2006	PRJEB9013
*A. veronii*	BVH 25b	3	Respiratory tract	Saint-Brieux, France, 2006	PRJEB9014
*A. veronii*	BVH 44	4	Wound	Périgueux, France, 2006	–
*A. hydrophila*	BVH 45	4	Wound	Périgueux, France, 2006	–
*A. media*	ADV 137a	5	Respiratory tract	Montpellier, France, 2010	–
*A. veronii*	ADV 137b	5	Respiratory tract	Montpellier, France, 2010	–
*A. sanarellii*	CAH 171	6	Wound	Cahors, France, 2013	–
*A. veronii*	CAH 172	6	Wound	Cahors, France, 2013	–
*A. veronii*	BVH 26b	–	Wound	Saint-Brieux, France, 2006	PRJEB9015
*A. dhakensis*	BVH 28b	–	Wound	Reunion island, France, 2006	PRJEB9016
*A. media*	BVH 40	–	Stools	Vannes, France, 2006	PRJEB9017
*E. coli*	OP50	–	–	–	–

### Typing methods

Pulsed-field gel electrophoresis (PFGE)-restriction fragment length polymorphism (RFLP) analysis was performed as previously described (Roger et al., 2012). DNA was digested using 40 U of *Swa*I (New England BioLabs, Hertfordshire, United Kingdom). A lambda concatemer (Biolabs) was used as the size standard for gel electrophoresis, and the PFGE profiles were compared visually.

### Bacterial growth rates and infection ratios

Growth curves were performed using a microplate reader (TECAN infinite 200) over 16 h. Complementary colony counts were performed to validate the ratios of the strains used in co-infections by either direct colony counts or by using TS agar containing 50 μg/ml cefalotin, as appropriate. Ratios were determined in the inoculum (on day 1) and in the bacterial lawn (on day 0 and day 3). As a result of small differences in the inocula, a ratio of 1:1 was defined as any ratio within the range of 1:3 to 3:1. Bacterial suspensions outside of this range were excluded from the analysis.

### Nematode killing assay (*C. elegans* model)

Assays were performed using the *C. elegans fer-15* conditional sterile mutant, which was provided by the Caenorhabditis Genetics Center. These worms are fertile at 15°C and sterile at 25°C. Experiments were performed as described elsewhere (Kurz et al., 2003). After a hypochlorite-bleach treatment, Fer-15 eggs produced at 15°C were spread on nematode growth medium (NGM) plate seeded with an *E. coli* OP50 lawn (Couillault and Ewbank, 2002) and incubated at 25°C for two days to yield a population of synchronous L4 stage sterile worms. NGM agar plates (60 mm) were inoculated with 50 μl of a single or mixed bacterial culture that was grown in TSB for 6 ± 1 h and incubated at 37°C for 18 ± 2 h. Plates were then equilibrated to room temperature, and L4 stage worms were spread on the lawn of bacteria (50–60 nematodes per plate). Dead and live worms were counted daily over 20 days using a stereomicroscope (Olympus SZH-ILLD). A worm was considered dead when it no longer responded to touch. Worms that were caught on the wall of the plate were excluded from the analysis. The assay was repeated at least three times for each condition, with the exception of the inoculum ratios analysis (one experiment). Time to death was recorded for each worm, and the time required to kill 50% of the worm population (TD_50_) was determined. Strains were considered a weak worm killer when TD_50_ was ≥7 days and a strong worm killer when TD_50_ was < 7 days, as previously described (Diard et al., 2007).

### Study strategy

**Figure 1:**
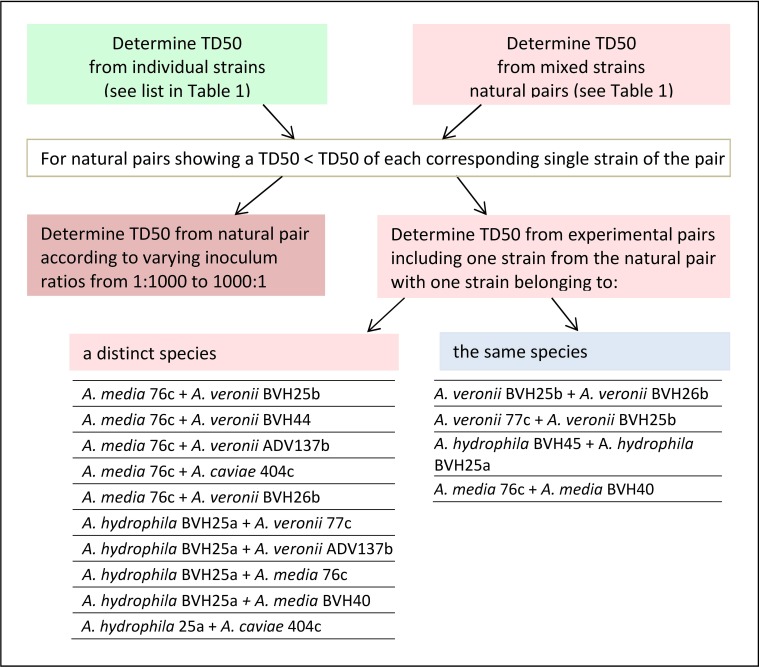
**Flowchart showing the sequential nematode killing assays (NKA)**. The median time to worm death (TD_50_) determinations were performed for single and paired strains according to the type of pair (naturally recovered or experimentally associated), and, for experimental pairs, according to the type of species pairing (same species or different species).

In this work, a “natural” pair refers to two distinct *Aeromonas* strains that were recovered from one single clinical sample (Table 1). An “experimental” pair denotes strains of clinical origin that were not isolated from the same sample and that belonged either to the same or to different species. The design of this study is shown in Figure 1, as follows: (i) the TD_50_ was determined for each strain individually and for the naturally occurring pairs, as indicated by the clinical sample number (Table 1). (ii) For a natural pair that showed a synergistic effect (i.e., a worm exposure to pairs resulting in time to death and a TD_50_ that are significantly less than the time to death and TD_50_ for each individual strain), the additional TD_50_s were determined after varying the inoculum ratio of the two associated strains from 1:1 to 1:1000. (iii) TD_50_ values were also determined for experimental pairs. Experimental pairs were designed to establish whether the effect was pair-specific. These included one strain from one synergistic natural pair and either a strain from another synergistic naturally occurring pair or a weak worm killer strain that belonged to a different species or to the same species (Figure 1). All experiments using strain pairs were performed after ensuring that the inoculum ratio was 1:1 for killing assays, except for experiments that studied the effect of the inoculum ratio on synergy.

### Virulence factors and comparative genomics

WGS were obtained for seven strains (Table 1), as previously described (Colston et al., 2014). Genome sequencing was performed at the Microbial Analysis, Resources and Services (MARS) facility at the University of Connecticut (Storrs, USA) using an Illumina MiSeq benchtop sequencer after preparing libraries from the genomic DNA using a Nextera XT DNA sample preparation kit (Illumina, San Diego, CA). Paired-end reads were trimmed and assembled into scaffolded contigs using a *de novo* assembler of CLC Genomics Workbench version 6.1.5 (CLC-bio, Aarhus, Denmark). The average genome coverage ranged from 44x to 130x, and the number of scaffolded contigs from 35 to 137, with an average of 83. Genomic contigs were annotated using the RAST annotation server to identify RNAs and protein-coding genes (Overbeek et al., 2014). The draft genomes were queried for genes encoding known virulence factors using either the translated sequences of the validated subset in UniProt (Swiss-Prot) or the annotated genes of the previously sequenced *Aeromonas* obtained from the GenPept or TrEMBL databases. Sequence comparisons of translated open reading frames were performed using BLASTP, and proteins with amino acid sequence similarities ≥65% and E-values ≤ 10^−10^ were considered to be homologs (Altschul and Lipman, 1990).

### Statistical analysis

Data were analyzed using GraphPad Prism version 5.00 for Windows (GraphPad Software, San Diego, CA). Descriptive statistics from the nematode killing assays included survival curves, TD_50_, and median TD_50_ with interquartile ranges (IQR). Data were analyzed both in terms of their survival curve and TD_50_. Briefly, data analysis was performed in two steps. First, comparisons of survival curves obtained within each experiment were performed using Log-rank tests with Bonferroni's correction to determine whether the survival observed with the pair was shorter than with both single strains. Second, data from distinct experiments were analyzed on the basis of TD_50_s comparisons. Difference in TD_50_s between the groups (either species or paired vs. single strains data) were assessed using Kruskal-Wallis tests or the Mann-Whitney tests where appropriate, with Bonferroni's correction. When the Bonferroni's correction was applied, the *P*-values were transformed accordingly. A *P*-value of ≤ 0.05 was considered significant (Supplemental Tables 1, 2).

### Accession numbers

The nucleotide sequences determined in this study were deposited into the GenBank database using the following accession numbers: KR140070—KR140076 (*gyrB*) and KR140077—KR140083 (*rpoB*). The WGS determined in this study were deposited in the European Nucleotide Archive (ENA) database (Table 1). The draft genomes sequenced and used in this study are also available for query and download at: http://aeromonasgenomes.uconn.edu.

## Results

### Strain characteristics

Strains included in all but one of the natural pairs belonged to different species (Table 1) and showed marked differences at the nucleotide level in *gyrB* and *rpoB* sequences (varying from 44 to 62 and 24 to 30 nucleotides, respectively). The 2 strains involved in one pair that belonged to the same species had different *gyrB* and *rpoB* sequences (a difference of 18 nucleotides and 1 nucleotide, respectively), and different PFGE patterns were observed for each member of this pair (data not shown). All strains, including those in natural pairs and those in experimental pairs, showed similar growth rates, with the following exceptions. Strain BVH28b quickly overgrew its paired strain. For example, when BVH28b was paired with strain BVH25a, these strains were counted at ratios of 5:1 and 10:1 within 7 h. Strain BVH45 overgrew strain BVH44 (at a ratio of 10:1 at day 3). Consequently, strain BVH28b was excluded from subsequent experimental paired analyses, and the natural pair BVH44+BVH45 could not be evaluated. Hence, only five natural pairs were studied.

### Single-strain killing assays

**Figure 2:**
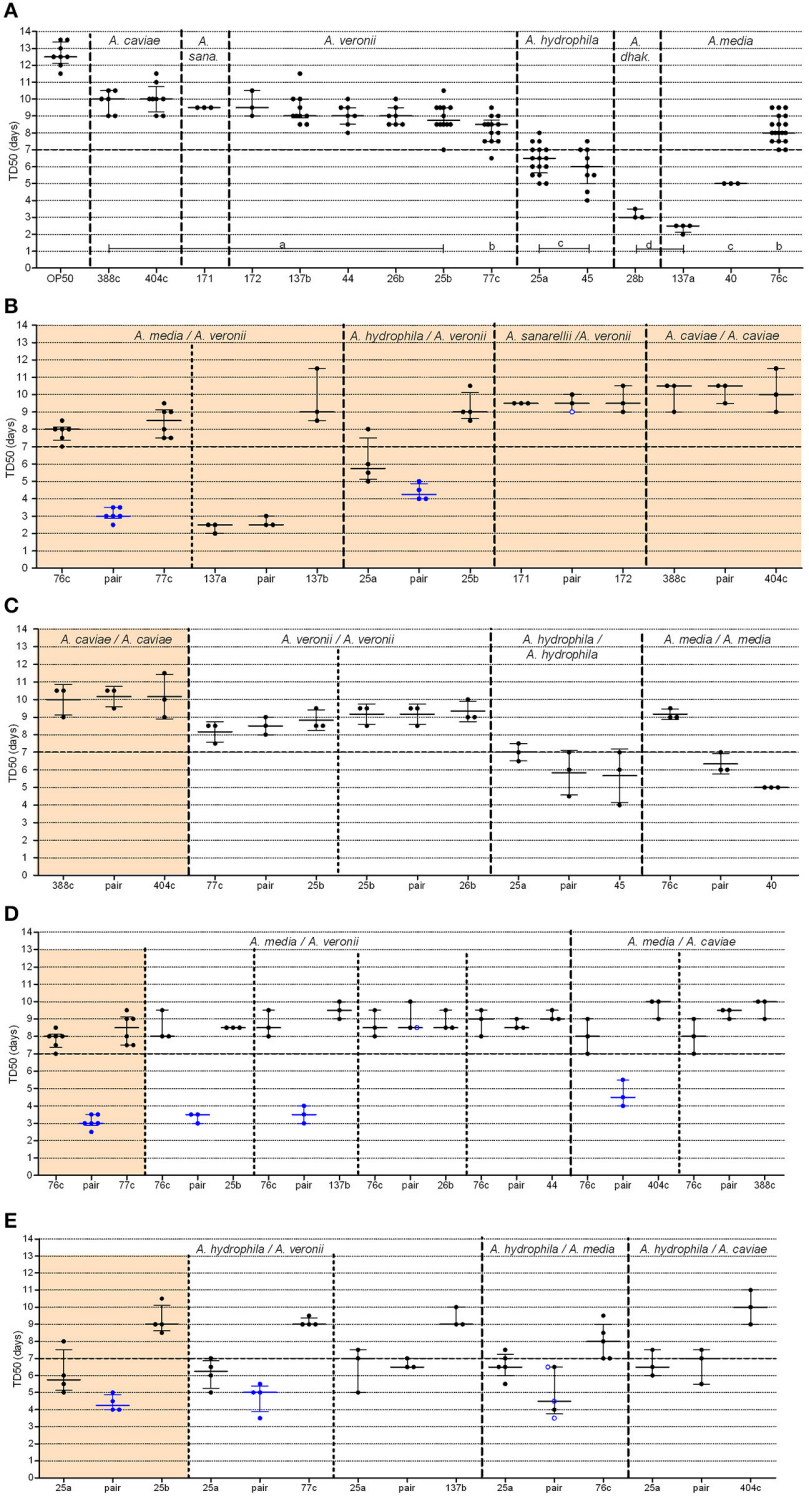
**Graphical representation of median time to worm death (TD_50_) obtained from strains administered alone or in pairs**. For clarity, the BVH, CAH, and ADV strain naming prefixes have been omitted. **(A)** Single strains (*A. dhak*: *A. dhakensis; A. sana: A. sanarelli*); **(B–E)** Strains in pairs, including: **(B)** natural pairs; **(C)** experimental pairs with strains belonging to the same species; **(D,E)** experimental pairs with strains from distinct species including either strain 76c **(D)** or strain BVH25a **(E)**. Each point represents the TD_50_ from one experiment and bars indicate the corresponding medians and interquartile ranges (IQR) of the TD_50_s. **(A)** Shows the plots (TD_50_) for each single strain that were obtained in all experiments. Light orange background sections correspond to data from natural pairs (i.e., both were recovered from a single clinical sample). Every section corresponds to pairs and single strains performed within the same assays to limit experimental variation. Mann-Whitney tests [or Kruskal-Wallis tests for data from **(A)**] with Bonferroni's correction were used for TD_50_s analysis. Log-rank tests with Bonferroni's correction were used for survival curves, as detailed in Supplemental Table 2. A *P*-value < 0.05 indicated that data were significantly different from each other. Single strain aeromonads **(A)** with significantly different TD_50_ values from each other are indicated by lowercase letters (**A**, a–d), and the corresponding statistics are detailed in Supplemental Table 1. Blue points **(B–E)** indicate pairs with (i) a shortened TD_50_ compared to both of the TD_50_ values of the corresponding single strains and (ii) shorter survival curves compared to both of the curves of the corresponding single strains. Black points indicate pairs with no difference both (i) in TD_50_ and (ii) in survival curves with the values of the corresponding single strains. Blue ringed white points indicate pairs (i) with shorter survival curves compared to both of the curves of the single strains but (ii) with no difference in TD_50_s compared to both of the TD_50_ values of the corresponding single strains.

Aeromonads in this study killed 50% of the *C. elegans* population within several days of exposure (range: 2.3–10.0 days, Figure 2A). Most of the TD_50_s were >7 days (in 10 out of 15 strains) or between 5 and 7 days (in 3 strains), indicating that most of the evaluated strains were weak worm killers. Two strains (one *A. media* strain, ADV137a, and one *A. dhakensis* strain, BVH28b) were much more virulent than the others, with median TD_50_ values of 2.5 days (IQR, 2.13–2.5 days) (*P*-value = 0.012) and 3.0 days (IQR, 3.0–3.5 days), respectively (*P*-value = 0.031) (Supplemental Table 1). Despite the very small number of strains used in this study, the TD_50_s was consistently similar within a species (e.g., all but one *A. veronii* strains displayed similar (*P*-value = 0.54) medians TD_50_ of 9.0 days (IQR, 8.5–9.5), the last strain (77c) displayed slightly shorter TD_50_s, with a median of 8.5 days (IQR, 7.5–8.8 days), *P*-value = 0.003), with the major exception of *A. media* (Supplemental Table 1). The three *A. media* strains displayed different TD_50_s (*P*-value = 0.004).

### Natural pair killing assays

In three of the five studied natural pairs, there were no significant difference in the TD_50_ values or in the survival curve observed between the individual strain and the cognate co-infection experiments (Figure 2B, Supplemental Figure 1). *A. caviae* 404c and *A. caviae* 388c exhibited similar virulence phenotypes when tested together (median TD_50_ of 10.5 days; IQR, 9.5–10.5 days) or alone (median TD_50_ of 10.0 days, IQR of 9–10.5 days (*P*-value = 0.75) and median TD_50_ of 10.0 days, IQR of 9.3–10.8 days), (*P*-value = 0.49), respectively) (Supplemental Table 2). The same behavior was observed with the strains *A. sanarellii* CAH171 and *A. veronii* CAH172 and their corresponding pairing, with no significant difference in TD_50_s. For the strains *A. media* ADV137a and *A. veronii* ADV137b, the mixed infection resulted in virulence that rose to the level of the strong worm killer, *A. media* ADV137a. There was no significant difference both in survival curves (*P*-values between 0.14 and 0.55, Supplemental Table 2) and in TD_50_s (*P*-value = 0.50) between the pair and ADV137a (Figure 2B, Supplemental Figure 1). At the opposite end of the spectrum, the virulence of the pairing including *A. media* 76c and *A. veronii* 77c was significantly higher (median TD_50_ of 3.0 days, IQR 3–3.5 days) than either strain 76c alone [median TD_50_ of 8.0 days (IQR, 7.5–9.0 days), *P*-value = 0.0004] or strain 77c alone [median TD_50_ of 8.5 days (IQR, 7.5–8.7 days), *P*-value = 0.0010]. The difference was also significant when comparing the survival curves (all *P*-values < 0.0001). A similar synergistic effect was observed for the pair including *A. hydrophila* BVH25a and *A. veronii* BVH25b compared to the effect of each individual strain (Figure 2B, Supplemental Figure 1). The TD_50_ was lower for the pair (median TD_50_ of 4.5 days, IQR, 4.0–5.5 days) than for either strain BVH25a alone (median TD_50_ of 6.5 days, IQR, 5.6–7.0 days) (*P*-value = 0.03) or strain BVH25b alone (median TD_50_ of 8.7 days, IQR, 8.5–9.5 days), (*P*-value = 0.004) alone. The difference was also significant when survival curves were compared (*P*-values ≤ 0.002).

**Figure 3:**
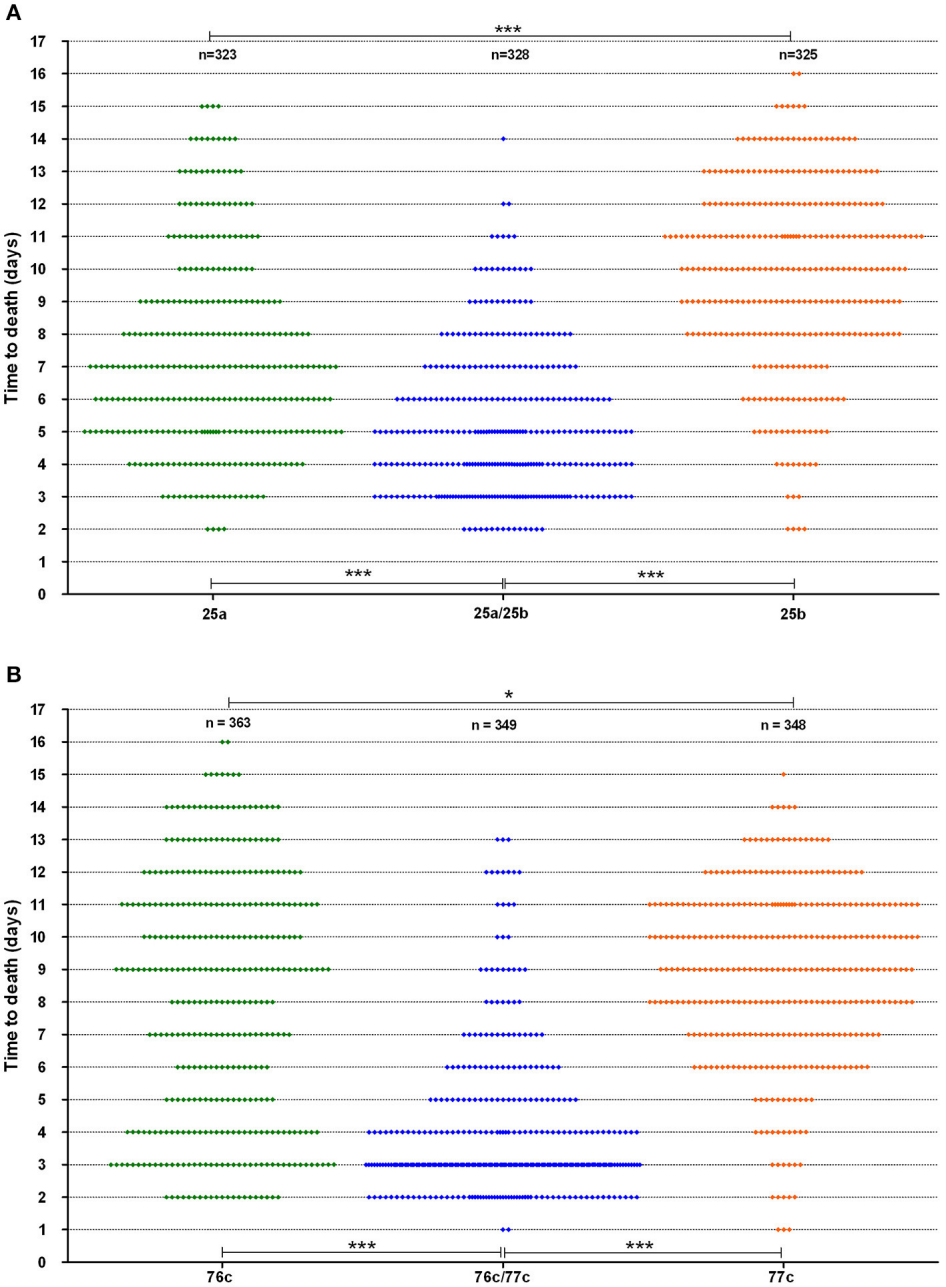
**Worm survival distribution (data from six experiments)**. **(A)** The BVH25a and BVH25b strains administered alone or in association and **(B)** The 76c and 77c strains administered alone or in association. Each plot represents one worm. Log-rank tests with Bonferroni's correction were used to compare distribution of worm death. A *P*-value < 0.05 indicated that populations were significantly different from each other. For both pairs, *P*-values of worm survival with the pairs were < 0.0001 (^***^) when compared to the worm survival observed with the corresponding single strains. Worm survival with the single strains were different from each other with a *P*-value of 0.01 (^*^).

The time to death in the worms was analyzed for strains with a synergistic virulence phenotype (Figure 3). Interestingly, strain 76c, considered a weak worm killer based on its TD_50_ values (median TD_50_ of 8.0 days, IQR of 7.5–9 days), killed 31% of the worm population within five days, as indicated by the bimodal time to death distribution (Figure 3B). Worms challenged with strain 77c, also a weak worm killer, exhibited a unimodal distribution in time to death that was centered at 8.5 days (Figure 3B), and only 12% of the overall population was killed within five days. Worm exposure with strains 76c and 77c resulted in increased virulence, with 78% mortality within five days, and the shape of the distribution of time to death in worms killed in 5 days or less was analogous to the one observed for the 76c alone. Time to death distributions were comparable when worms were fed on either old and fresh bacterial lawns or when half the number of worms were challenged (data not shown), suggesting that the bimodal distribution pattern was not due to the age of the bacterial population in the lawn or to the amount of bacterial food. Altogether, these results show that while strain 76c alone is capable of quickly killing a substantial number of worms, when combined with strain 77c, the total virulence is strengthened. For strains BVH25a and BVH25b, the distribution of the time to death was unimodal (Figure 3A). When mixed, the distribution shape was analogous to that of BVH25a, but it was lower and showed less variance.

### The effect of the inoculum ratio on synergy

**Figure 4:**
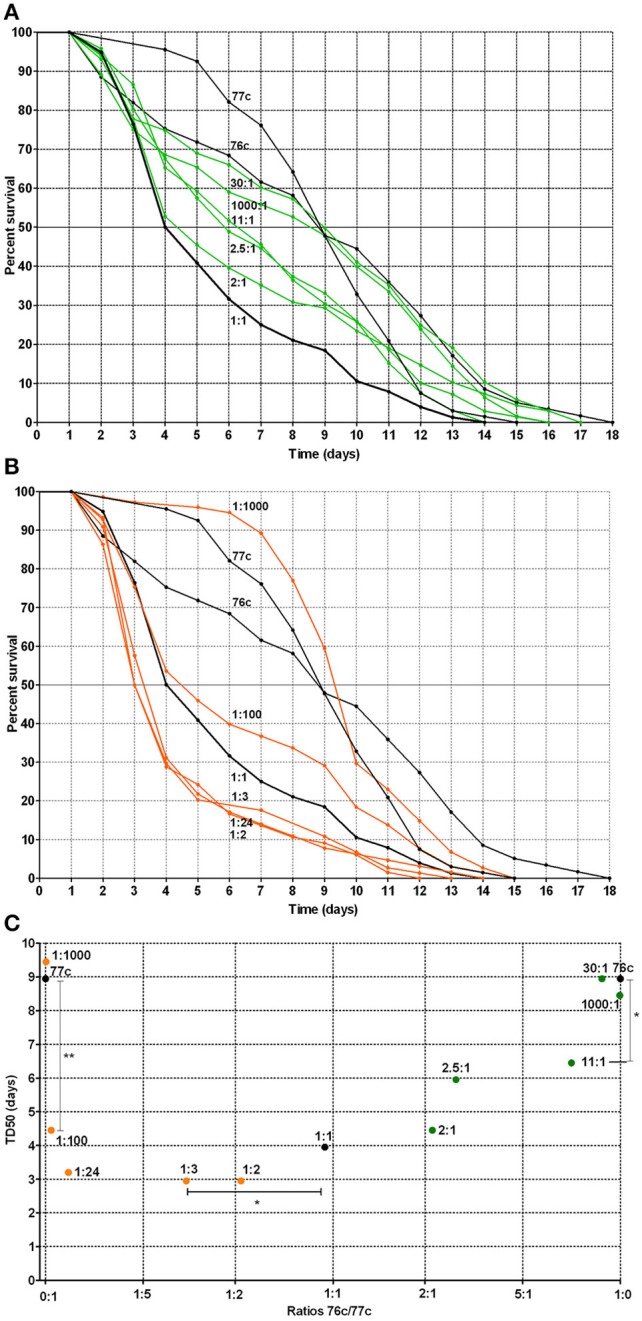
**Worm survival according to mixed infection with strains 76c and 77c at varying inoculum ratios (ratios from 1:1000 to 1000:1, with 76c and 77c being presented as the first and second strain of the ratio, respectively)**. **(A)** Orange curves correspond to worm survival curves for strains in which 77c prevailed (ratios from 1:2 to 1:1000). Black curves (1:0, 0:1, and 1:1) correspond to worm survival curves for the single strains 76c and 77c and for mixes in which strains 76c and 77c were included at equal density, respectively. **(B)** Green curves correspond to worm survival curves for mixes in which strain 76c prevailed (ratios from 2:1 to 1000:1). Black curves (1:0, 0:1, and 1:1) correspond to worm survival curves for the single strains 76c and 77c and for mixes containing strains 76c and 77c in equal density, respectively. **(C)** Median time of worm death (TD_50_), according to the relative frequency of 76c within the mix. The statistical analysis performed was log-rank tests with Bonferroni's correction. A *P*-value < 0.05 indicated that survival curves were significantly different from each other, as presented in Supplemental Table 2. ^*^Indicates a *P*-value between 0.01 and 0.05; ^**^ indicates a *P*-value between 0.001 and 0.009.

For the natural pairs that exhibited a synergistic effect, we next evaluated the time to death using varying ratios of the strains to determine the optimal ratio for synergistic virulence. We focused on the pair 76c+77c (76c:77c, Figure 4A and 77c:76c, Figure 4B) because the change in TD_50_s was the highest for this pair. Compared with single strains 76c and 77c, virulence was enhanced when strains 76c and 77c were inoculated at ratios between 11:1 (*P*-value = 0.040) and 1:100 (76c:77c) (*P*-value = 0.009). The shortest TD_50_ occurred when strain 77c predominated (ratios 1:2–1:24, see Figures 4B,C), although synergy was marked at ratios of 1:100 and 1:1.

### Experimental pair killing assays

The strains in natural pairs that exhibited increased virulence (pair 76c+77c and pair BVH25a+BVH25b) belonged to distinct species. To investigate whether this synergistic effect was pair-specific and to determine whether there was a species effect, TD_50_ was measured for experimental pairs that included one of the strains involved in a natural pair with synergistic virulence and another strain (Figure 1). None of the four experimental pairs that contained strains belonging to the same species showed increased virulence compared to the virulence of the corresponding single strains (Figure 2C, Supplemental Table 2), which is comparable to what was observed for the natural pair *A. caviae* 388c+*A. caviae* 404c.

Different results were obtained in experimental pairings that consisted of strains from different species. A synergistic effect comparable to the effect observed in the 76c+77c pairing was observed when *A. media* strain 76c was paired with three other strains, including the *A. veronii* strains BVH25b and ADV137b, and the *A. caviae* strain 404c (TD_50_ 3.4–4.7 days, Figure 2D and Supplemental Table 2). Pairs exhibited shortened TD_50_s (all *P*-values between 0.001 and 0.040). Interestingly, the shape of the distribution for time to death for these experimental pairs was comparable to that of the natural pair 76c+77c (data not shown). These results suggested that the strain 76c ability to quickly kill worms is strengthened by the companion strain. No synergistic effect was observed when strain 76c was paired with *A. veronii* BVH44, BVH26b or *A. caviae* 388c (TD_50_ 8.4–9.0 days, all *P*-values ≥ 0.09, Figure 2D and Supplemental Table 2). A synergistic effect comparable to the effect observed for the BVH25a+BVH25b pairing was observed when the *A. hydrophila* strain BVH25a was combined with the *A. veronii* strain 77c (TD_50_ 4.7 days, *P*-values < 0.02). In combination with the *A. media* strain 76c, experiments gave conflicting results, and three experiments showed a synergistic effect in survival curve comparisons (*P*-values = 0.02) while 2 experiments did not (Figure 2E, Supplemental Table 2). No significant synergistic effect was observed when BVH25a was paired with either the *A. veronii* strain ADV137b or the *A. caviae* strain 404c (TD_50_ 6.7–7.3 days, all *P*-values ≥ 0.14, Figure 2E).

### Virulence factor genes and quorum sensing (QS) genes

To investigate whether the differences in virulent behaviors observed following exposure to single strains or pairs could have resulted from the presence of previously described virulence factors, genome contents were analyzed with a focus on genes known to be virulence factors. Moreover, the inoculum ratio results revealed an effect on synergy, which suggested an effect at the population level. Because virulence and population behaviors are related to quorum sensing, we sought to determine whether the strains contained quorum sensing systems (QS). Draft genomes (length of 4.58–5.1 Mbp) of seven of the strains were screened for over 50 genes corresponding to approximately 40 different gene loci known to be associated with virulence and QS (Table 2). All of the strains contained a wide array of virulence-associated genes, and many of these genes were present in every genome (Table 2). All of the draft genomes also encoded the QS-associated genes derived from the three systems described in *Aeromonas*; these included the LuxRI, LuxS, and QseBC systems. A few virulence-associated genes were not found in any of the genomes (e.g., shiga-like toxin and sialidase). The type six secretion system (T6SS)-associated genes were present in all genomes except strains 76c and 77c. Interestingly, in both the 76c+77c and BVH25a+BVH25b natural pairings, only one strain from each pair carried the type three secretion system (T3SS)-associated genes. This trend was not observed in experimental pairs that displayed synergistic virulence. We failed to identify a virulence pattern that could be correlated to an observed synergistic behavior.

**Table 2:** **Presence or absence of protein-coding genes (CDS) in the genomes of seven strains analyzed in this study**.

**Putative protein CDS**	***A. hydrophila* BVH25a**	***A. veronii* BVH25b**	***A. veronii* BVH26b**	***A. dhakensis* BVH28b**	***A. media* 76c**	***A. veronii* 77c**	***A. media* BVH40**
**PUTATIVE TOXINS AND EXOENZYMS**							
Aerolysin, Enterotoxin cytotoxic Act **(Q06304)**	−	+	+	+	−	+	−
Hemolysin Ahh1 **(P55870)**	+	−	−	+	−	−	−
Heat-labile cytotonic enterotoxin, lipase (A0KEH6)	+	+	+	+	+	+	+
Heat-stable cytotonic enterotoxin (Q8VRN3)	+	−	−	−	−	−	−
Shiga-toxin 1 subunit A (E2DQN2)	−	−	−	−	−	−	−
Shiga-toxin 2 subunit A (E2DQN6)	−	−	−	−	−	−	−
Extracellular deoxyribonuclease **(P39658)**	+	+	+	+	+	+	+
U32 family collagenase (A7M6D1)	+	+	+	+	+	+	+
Elastase (Q9RMM8)	+	+	+	+	+	+	+
Enolase **(A0KGH3)**	+	+	+	+	+	+	+
S8 familly serine protease (A4SNU7)	+	+	+	+	+	+	+
Exotoxin A (T0P5W3)	−	−	−	+	−	−	−
Phospholipase GCAT **(P10480)**	+	+	+	+	+	+	+
Sialidase (R4VB69)	−	−	−	−	−	−	−
Aminopeptidase N (A0KKL2)	+	+	+	+	+	+	+
Tox-R activated lipoprotein TagA (A4SQY1)	+	+	+	+	+	+	+
Ribonuclease R (B2L1Z1)	+	+	+	+	+	+	+
**SIDEROPHORES**							
Isochorismate synthase **(P23300)**^a^, (F4DG47)^b^	+^a^	+^b^	+^b^	+^a^	+^a^	+^b^	+^a^
TonB-dependent siderophore receptor (*YP_856500.1*)^a^, (F4DG48)^b^	+^a^	+^b^	+^b^	+^a^	+^a^	+^b^	+^a^
**TYPE 3 SECRETION SYSTEM (T3SS) AND EFFECTORS**							
Inner membrane channel protein AscV (A4SUH2)	+	−	+	+	−	+	−
Needle protein AscF (A4SUF6)	+	−	+	+	−	+	−
ADP-ribosyltransferase toxins AexT **(Q93Q17)**^a^ and AexU **(D5LUP3)**^b^	+^b^	−	+^b^	+^b^	−	+^a, b^	−
**TYPE 6 SECRETION SYSTEM (T6SS) AND EFFECTORS**							
Sigma 54-dependent transcriptional regulator VasH (Q0PZG3)	+	+	+	+	−	−	+
Hemolysin-coregulated protein Hcp (Q6TP03)	+	+	+	+	−	−	+
**APPENDAGES**							
**P fimbriae** Outer membran fimbrial usher protein (A0KFM8), Periplasmic fimbrial chaperone (F4D857)	+	+	−	+	−	+	−
**Type IV fimbriae** Type IV pilus assembly proteins TapB **(P45792)** and TapC **(P45793)**	+	+	+	+	+	+	+
**Mannose-sensitive hemagglutinin pilus (MSHA)** MSHA biogenesis protein MshL (F4DEK1) and MSHA pilin protein MshB (H9NKW0)	+	+	+	+	+	+	+
Flp pilus assembly protein FlpC (A4SPU9)	−	+	−	−	−	−	−
**Polar flagellum** Flagellin protein FlaA (Q9R9R9), Flagellar hook-associated protein FliD **(Q9R9R6)** and FliS (A0KIY7)	+	+	+	+	+	+	+
**Lateral flagellum** Lateral Flagellin LafA **(Q93TL9)**	+	−	+	+	−	+	+
**SURFACE POLYSACCHARIDES**							
Polysaccharide export lipoprotein Wza (A0A068FZJ7), Cytoplasmic tyrosine phosphatase Wzb (*WP024942466*), and Tyrosine kinase Wzc (A0A023RLX8)	−	+	−	−	+	−	−
Capsular polysaccharide biosynthesis protein (F4DBW0)	−	+	+	+	−	+	−
Lipid A core-O-antigen ligase (A0KM76)	+	+	+	+	+	+	+
LPS-assembly protein LptD **(A0KGU1)**	+	+	+	+	+	+	+
**BINDING PROTEINS**							
Surface layer protein VapA **(P35823)**	+	−	−	−	−	−	−
Major adhesin Aha1 (*ABC54614.1*)	+	+	+	+	+	+	+
**QUORUM SENSING**							
**Type 1 Quorum sensing** Acyl-homoserine-lactone synthase AhyI **(Q44058)**, Transcriptional activator protein AhyR **(P0A3J5)**	+	+	+	+	+	+	+
**Type 2 Quorum sensing** S-ribosylhomocysteine lyase LuxS **(A0KG57)**	+	+	+	+	+	+	+
**Type 3 Quorum sensing** Two-component system response regulator QseB (G4XZM6), Sensory histidine kinase QseC (G4XZM7)	+	+	+	+	+	+	+
**HYPOTHETICAL PROTEIN**							
AHA_0904 (*YP_855447.1*)	+	+	+	+	+	+	+

*Accession numbers from TrEMBL, Swiss-prot (bold), or GenPept (italic) databases. When two different types of protein with the same annotation were identified as reference sequences in the databases, homologous CDS were arbitrarily distinguished by superscript letters (a and b). For each strain harbouring one of these genes, the CDS with the most significant E-values is indicated by the corresponding superscript letter*.

## Discussion

Despite the relatively small number of pairs tested, our results provide experimental support for the role of bacterial synergy in *C. elegans* infection model and several implications related to infection and virulence determinants.

### Co-infection

With current changes of paradigm in microbiology during the microbiota and pathobiome era (Vayssier-Taussat et al., 2014), polymicrobial infections are growing more interesting. In the case of aeromonosis, the high frequency of co-infection by two or more strains (Lamy et al., 2009; Figueras and Beaz-Hidalgo, 2015) and our current results suggest the potential existence of interactions between bacteria, which could include either cooperation between bacteria that results in a pathogenic outcome or competition between bacteria for a common habitat and resources. The frequency of aeromonad co-infections (5–10%) is probably underestimated, and this number should be considered to be a minimum because multi-strain cultures can be missed when colony morphotypes are similar (normally, a single colony is taken from the recovered isolates for identification). Besides, in the natural symbiotic system of the leech digestive tract, *A. veronii* bv. sobria and *Mucinivorans hirudinis* showed synergistic bacterial cooperation in mixed microcolonies containing both species, where colonies were observed to be consistently larger than the microcolonies of each single species (Kikuchi and Graf, 2007). This is an interesting report supporting bacterial interaction in virulence because beneficial symbiosis and pathogenesis determinants are often similar (Hentschel et al., 2000; Hussa and Goodrich-Blair, 2013; Pérez-Brocal et al., 2013).

Considering the pathogenic mechanisms involved in bacterial associations, there is increasing evidence that some synergistic relationships between pathogens involved the production and utilization of specific virulence factors and exoproducts, showing that some bacteria benefit from others, and leading to an overall intensification of their virulence (Crane et al., 2006; Lo et al., 2011; Ramsey et al., 2011; Korgaonkar et al., 2013). For instance, hemolysis and cytolysis by *S. aureus* were increased when *S. aureus* was grown with CAMP factor-producing *Propionibacterium acnes*, and *P. acnes* exacerbated *S. aureus*-induced skin lesions *in vivo* (Lo et al., 2011)*. P. aeruginosa* used peptidoglycan shed by Gram-positive bacteria as a cue to stimulate production of multiple extracellular factors that possess lytic activity against cells (Korgaonkar et al., 2013).

Over the past decade, animal models have been successfully used to investigate single strain infections (e.g., Kurz et al., 2003; Sifri et al., 2005; Lavigne et al., 2008; Bogaerts et al., 2010; Mellies and Lawrence-Pine, 2010; Merkx-Jacques et al., 2013; Chen et al., 2014), and they may also be powerful systems for studying infectious synergy *in vivo*. Nematode and arthropod systems provide interesting models that are focused on the behavior of pathogens that face organized and differentiated tissues and innate immunity. The synergistic effect observed when uropathogenic *E. coli* and *Enterococcus faecalis* were associated corroborated the clinical concept proposing that increased virulence occurs in urinary tract infection when these two species are mixed (Lavigne et al., 2008). Hence, these data demonstrate that *C. elegans* is a realistic model for studying mixed infections in humans.

### *Aeromonas* virulence in the *C. elegans* model

In this study, a low percentage of *Aeromonas* isolates recovered from natural human infections were highly virulent when tested individually (2 out of 15). Interestingly, one of these strains belongs to *A. dhakensis*, a species for which a higher virulence was described (Chen et al., 2014). The second strain belongs to a species rarely associated with disease in humans: *A. media* (Lamy et al., 2009; Janda and Abbott, 2010). Under the experimental conditions used in this study, we observed some rapid lysis in cadavers by strain *A. dhakensis* BVH28b, a rare event in our experience that was also observed by Couillault and Ewbank (2002) with some *Aeromonas* strains. The mechanisms used by *Aeromonas* to kill worms remain to be determined.

The major result of this study is the enhanced virulence observed during aeromonad co-infection in *C. elegans*, in that some specific pairs of strains were less virulent individually than they were when paired. Strong worm killing behavior was more frequently observed when strains were in pairs than when they were used alone, suggesting an increase in pathogenic behavior when multiple aeromonads were present.

Our study suggests that synergy occurred only between strains from different species. The reason for this increase in virulence and the mechanism by which it was enhanced by other strains that belonged to different species remain unclear. Several hypotheses can be tested in future studies. On the one hand, increasing evidence related to signaling and cooperation between distinct and unrelated species supports a scenario involving bacterial cooperation (Crane et al., 2006; Lo et al., 2011; Ramsey et al., 2011; Peters et al., 2012; Korgaonkar et al., 2013). At this stage, we cannot exclude the possibility that only one of the strains benefits by receiving signals and/or products from the other strain (Mellies and Lawrence-Pine, 2010; Lo et al., 2011; Ramsey et al., 2011; Korgaonkar et al., 2013). In such a case, the synergy would be frequency-dependent: when the producers/cooperators are more common, there are more opportunities to benefit from signaling or generated products (Diggle et al., 2007). This could explain why the virulence curves and TD_50_s changed according to inoculum ratios in an asymmetric manner (Figure 4C). In fact, the overall results first show that strain 76c is itself capable of quickly killing worms (Figure 3), but this ability was more easily expressed when strain 77c was predominate, suggesting that strain 76c benefited from the presence of strain 77c. Because the effect was reproduced with strain 76c paired with other strains (e.g., strains 137b, 25b, 404c) with the same level of virulence, the assumption that the ability to quickly kill is borne by strain 76c and is enhanced by the companion strain is at least to be considered. Second, results from inoculum ratios analyses show that if both strains 76c and 77c are able to enhance their virulence when associated with the other strain, strain 76c changes its behavior when the other strain is more common than does strain 77c [e.g., 1:100 compared to 2:1 curves (76c:77c), Figure 4C]. We cannot exclude that strain 76c better or earlier exploits goods produced by the other strain than strain 77c. However, this does not explain why synergy was observed only in strain pairs involving different species, unless we hypothesize that the genetic repertoires are complementary and enriched to favor cooperation when repertoires from more distantly related organisms are present than when they are closely related organisms. Because QS systems were found in all *Aeromonas* strains we tested, we hypothesize that QS is at least partly involved in the induction of the mechanisms that cause this enhanced virulence.

On the other hand, enhanced virulence may be a consequence of competition. Kin selection theory states that lower levels of relatedness lead to increased competition, which favors rapid growth to achieve greater relative success within host, and that higher bacterial growth rates lead to higher virulence (Franck, 1996; Pollitt et al., 2014). This could also explain why enhanced synergy is perceptible at ratios of 1:100, as differences in virulence phenotypes observed with varying ratio inoculums could result from competition (Figure 4C). However, the opposite hypothesis has also been proposed: a positive correlation between relatedness and virulence. This hypothesis is in contradiction with our results (West and Buckling, 2003). However, the picture could be blurred by the fact that the *Aeromonas* strains included in this work are close relatives even if they belong to distinct species because the genus *Aeromonas* is an expansive complex of closely related species (Roger et al., 2012). Whether enhanced virulence relies on cooperation or competition requires further study.

### Pathotypes, or bacterial cooperation, or both?

Many questions concerning the specifics of the pathogenicity of aeromonads remain unanswered. The idea of pathotypes has been proposed (Janda and Abbott, 1998, 2010; Joseph and Carnahan, 2000), but the level of evidence supporting this hypothesis remains low. Recently, Grim et al. (2013) have, based on a pair of strains recovered from a wound infection case, provided evidence for two pathotypes. However, the authors did not discuss any possible bacterial interaction that might have influenced the course of the infection (Grim et al., 2013). They defined a necrotizing pathotype based on the presence of a triad or quartet of outstanding virulence factors that included the T3SS AexT and/or AexU, the toxin Aerolysin (syn. Act), the T6SS and the ability to produce biofilm.

Although these findings are consistent with the hypothesis of there being more virulent subsets within each *Aeromonas* species, their results failed to completely clarify the picture of pathogenic/non-pathogenic strains (Grim et al., 2013, 2014). In this study, we found that one strain out of every synergistic natural pair harbored T3SS and aerolysin, but this association was not confirmed in all synergistic experimental pairs (e.g., pair 76c+BVH25b), and the pair 76c+BVH26b showed no synergistic effect despite the fact that BVH26b harbored *aexU* and aerolysin genes. In addition, T6SS-associated genes were not found in either of the strains in the synergistic natural pair 76c+77c, suggesting that the pathotype defined by Grim et al. is not generic to all aeromonads but is perhaps specific and limited to the rare necrotizing pathotype (Grim et al., 2013), and that this virulence panel fails to explain many other pathogenic behaviors.

Here, the most virulent strains or pairs did not necessarily contain the highest number of virulence genes or well described virulence factors. This reinforces the idea that virulence in *Aeromonas* results from a complex situation that is difficult to understand from only the genome contents of virulence-associated genes, and that environmental factors modulate virulence gene expression. The absence of well-described virulence factors in strong killer strains (e.g., T3SS, T6SS, capsule) suggests that either some of the virulent determinants in *Aeromonas* remain to be described, that aeromonads may use diverse strategies to induce virulence, that some of them do not rely on known or well-described virulence factors, or that phenotypes are different in the *C. elegans* model from the phenotypes observed in other animals.

In addition, in an attempt to document pathotypes resulting from adaptations to human ecology, we previously searched for genetic clusters that corresponded to ecologically distinct and virulent aeromonads (Roger et al., 2012). From a large collection of strains (195 strains, including 115 human clinical strains), we identified only a very small subsets of strains, although all of them were disease-associated and included in small clonal complexes, suggesting that adaptation to human ecology is, at best, rare. Our current results suggest a role for interactions between bacteria that enhance virulence, and virulence in aeromonads should be envisioned as a spectrum of host-microbe pathogenic mechanisms, microbe-microbe interactions, host immunity-mediated antimicrobial defenses, and environmental factors.

The two hypotheses, involving pathotypes and bacterial cooperation, are not exclusive or conflicting hypotheses. They may complement each other in determining pathogenicity, as bacterial pathogenicity may involve several strategies. Their combination, in addition to the diversity of available virulence factors, may explain why it is so difficult to determine the source of aeromonad virulence. Finally, in the light of these studies, our results provide new avenues for explaining several complex scenarios. Among them, we need to question whether mixed synergistic *Aeromonas* infections result from chance unfortunate encounters between complementary bacteria and a host, or from a more subtle system wherein two distinct pathogens form a unit with a selective advantage to specifically cause infection.

### Future perspectives

The current key challenges are to determine the precise mechanisms involved in these polymicrobial interactions. One of the hypotheses that deserves to be explored is the role of QS molecules in addition to interspecies signaling and bacterial cell to cell communications because they interact with innate immune functions, regulate virulence, and are involved in nematode infections (Sifri et al., 2005; Mellies and Lawrence-Pine, 2010) or in the indirect pathogenicity of some bacteria [e.g., polymicrobial otitis media (Armbruster et al., 2010)]. Moreover, the genomic sequences of all of the strains in this study contain QS systems (Table 2). Another hypothesis concerns cooperative goods and harmful toxin production.

Our study of *Aeromonas-Aeromonas* mixed infection was designed as a simple model that can be expanded to mixed infections with other genera, such as *Aeromonas-Staphyloccocus aureus* or *Aeromonas*-Enterobacteria. Beyond the initial test case of aeromonosis, this issue should be of interest to researchers of every disease in which mixed infections are likely in relation to their corresponding epidemiology, e.g., water-related infections, such as Legionnaire's disease and vibriosis (Coscollá et al., 2014).

## Author's contributions

Conceived and designed the study: TM, BL, designed and performed the acquisition of clinical isolate collection: MF, BL, Performed the microbial and *C. elegans* analyses: TM, Performed acquisition and analyses of whole genome data: SC, ET, JG, Analyzed and interpreted the data: TM, BL (microbial data), ET, SC (WGS), TM, BL (statistics), Drafted the paper: BL, helped to draft the manuscript: EJB, Critically revised the manuscript: EJB, JG, MF, TM, ET, SC. All authors read and approved the final manuscript.

### Conflict of interest statement

The authors declare that the research was conducted in the absence of any commercial or financial relationships that could be construed as a potential conflict of interest.

## Supplementary material

Supplemental Figure 1 **Worm survival curves obtained from strains administered alone or in pairs**. Assays were repeated three to six times for each condition. Log-rank tests with Bonferroni's correction were used to compare curves obtained within each experiment. A *P*-value < 0.05 indicated that survival curves from the pairing were significantly different from the curves of the corresponding single strains, as detailed in Supplemental Table 2. Green and orange curves correspond to survival curves for single strains. Blue curves correspond to survival curves of pairs that were significantly lower to both of the curves of the corresponding single strains (i.e., a synergistic virulence phenotype). Black curves correspond to survival curves of pairs that were not significantly lower to both of the curves of the corresponding single strains.

Supplemental Table 1 **Statistics used for comparison of single strain killing assays**.

Supplemental Table 2 **Statistics used for comparisons of pair killing assays data**.
