# Supplementary figures and images for: Exposure to pairs of Aeromonas strains enhances virulence in the Caenorhabditis elegans infection model
Source: Front Microbiol. 2015 Nov 4;6:1218. doi: 10.3389/fmicb.2015.01218 (PMC4631986; doi:10.3389/fmicb.2015.01218)

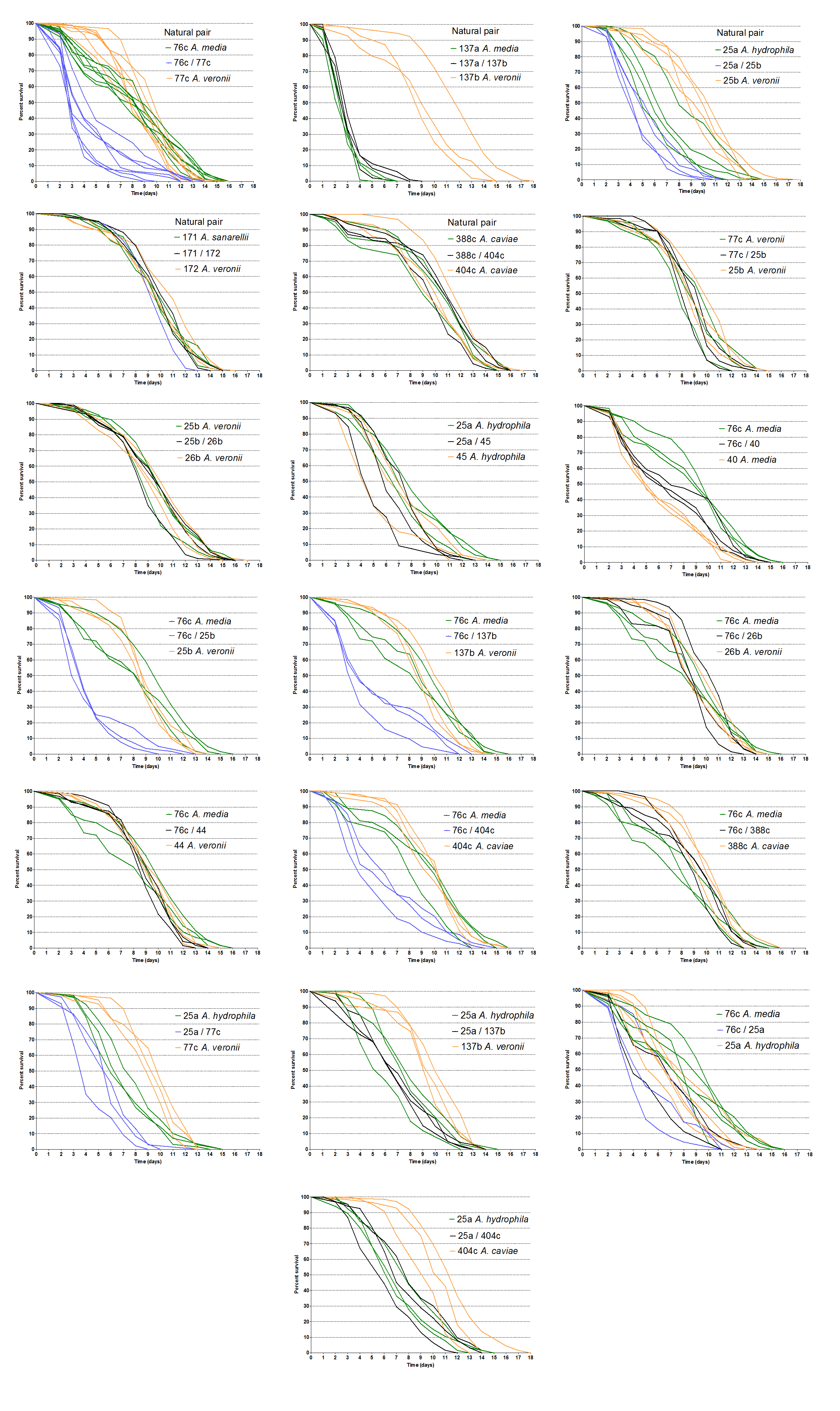

Supplement: Supplemental Figure 1 — Worm survival curves obtained from strains administered alone or in pairs. Assays were repeated three to six times for each condition. Log-rank tests with Bonferroni's correction were used to compare curves obtained within each experiment. A P-value < 0.05 indicated that survival curves from the pairing were significantly different from the curves of the corresponding single strains, as detailed in Supplemental Table 2. Green and orange curves correspond to survival curves for single strains. Blue curves correspond to survival curves of pairs that were significantly lower to both of the curves of the corresponding single strains (i.e., a synergistic virulence phenotype). Black curves correspond to survival curves of pairs that were not significantly lower to both of the curves of the corresponding single strains. [file Image1.JPEG]
