# Supplementary material for: Exposure to pairs of Aeromonas strains enhances virulence in the Caenorhabditis elegans infection model
Source: Front Microbiol. 2015 Nov 4;6:1218. doi: 10.3389/fmicb.2015.01218 (PMC4631986; doi:10.3389/fmicb.2015.01218)
Supplement: Supplemental Table 1 — Statistics used for comparison of single strain killing assays. [file Table1.PDF]

Supplemental Table 1: statistics used for comparison of single strain killing assays. The tests were performed to determine whether the *Aeromonas* single strains displayed different virulence phenotypes from each other. Comparisons were performed on TD_{50}s that were obtained from several experiments, by using Kruskal-Wallis tests or Mann-Whitney tests where appropriate with Bonferroni's correction. Comparisons of TD50s using Kruskal-Wallis or Mann-Whitney tests were preferred to log-rank tests given the number of curves and of different experiments. A *P* value <0.05 indicated that comparisons were statistically different (bold values). Results of the analysis are shown on Figure 2A.

Group of strains tested	*P* value	Comment
All *Aeromonas* strains (n=15)	**<0.001**	Strains displayed different virulence phenotypes from each other
Strains 388c, 404c, CAH171, CAH172, ADV 137b, BVH 44, BVH 26b, BVH 25b [a]	0.22	These strains displayed no difference in virulence from each other. These strains are indicated by the letter "a" in Figure 2A
Strains 388c, 404c, CAH171, CAH172, ADV 137b, BVH 44, BVH 26b, BVH 25b, and 77c [b]	**0.003**	Strain 77c displayed different TD_{50}s compared to those of the strains indicated by letter "a" in Figure 2A. This difference is indicated by the letter "b" in Figure 2A
All *A. veronii* strains, except strain 77c (i.e., CAH172, ADV 137b, BVH 44, BVH 26b, BVH 25b)	0.54	
A. media strains, *i.e.* strains ADV 137a, BVH40, 76c	**0.004**	*A. media* strains displayed different virulence phenotypes from each other
*S*trains BVH 25a, BVH 45, BVH 40	0.067	These strains displayed no different TD_{50}s from each other. These strains are indicated by letter "c" in Figure 2A
Strains BVH 28b versus ADV 137a	0.17	These strains displayed no different TD_{50}s from each other. These strains are indicated by letter "d" in Figure 2A
Strains 76c versus 77c	0.98	These strains displayed no different TD_{50}s from each other. These strains are indicated by letter "c" in Figure 2A
Strain BVH28b and *s*trains BVH 25a, BVH 45, BVH 40 (i.e., group "c")	**0.031**	Strains BVH28b and ADV137a displayed shorter TD_{50}s compared to TD_{50}s of the strains indicated as group "c"
Strain ADV137a and strains BVH 25a, BVH 45, BVH 40 (i.e., group "c")	**0.011**	

[a] group including strains from the species *A. caviae* and strains from the *A. veronii* with the exception of strain 77c

[b] group including strains from the species *A. veronii*
