# Supplementary material for: Exposure to pairs of Aeromonas strains enhances virulence in the Caenorhabditis elegans infection model
Source: Front Microbiol. 2015 Nov 4;6:1218. doi: 10.3389/fmicb.2015.01218 (PMC4631986; doi:10.3389/fmicb.2015.01218)
Supplement: Supplemental Table 2 — Statistics used for comparisons of pair killing assays data. [file Table2.PDF]

Supplemental Table 2: statistics used for comparisons of pair killing assays data. Comparisons were performed either on survival curves for each assay or on TD_{50}s that were obtained from several assays. Synergy of virulence was defined by a significantly lowered survival curve to both of the curves of the corresponding single strains and by shortened TD_{50} values to both of the TD_{50} values of the corresponding strains. A *P* value <0.05 indicated that comparisons were statistically different (bold values). For clarity, the BVH, CAH and ADV strain naming prefixes have been omitted. Statistics presented in text, unless specified, correspond to results of TD_{50}s analysis that including data from several assays.

			Survival curves comparisons						TD_{50}s comparisons	Comment
Datasets used for tests			Survival curves obtained within each assay						TD_{50}s obtained from several assays (3 to 6 TD_{50} values of per condition)	TD50s and survival comparisons were overall in accordance
Test used			Log-rank tests with Bonferroni's correction [b]						Mann-Whitney tests with Bonferroni's correction [c]	
Tests results per condition			*P* values						*P* values	
Series No.			1	2	3	4	5	6	All	
Strain pairing (Figure 2 B-E, Supplemental Figure 1)	76c/77c [a,c]	Pair vs 76c	**<0.0001**	**<0.0001**	**<0.0001**	**<0.0001**	**<0.0001**	**<0.0001**	**0.0004**	The pair displayed synergistic virulence phenotype
		Pair vs 77c	**<0.0001**	**<0.0001**	**<0.0001**	**<0.0001**	**<0.0001**	**<0.0001**	**0.0010**	
	137a/137b [a,c]	Pair vs 137a	0.39	0.55	0.14	-	-	-	0.50	Virulence phenotype of the pair equaled but did not exceed the level of the strong worm killer strain 137a. Absence of synergistic virulence phenotype (see Supplementary Figure 1).
		Pair vs 137b	**<0.0001**	**<0.0001**	**<0.0001**	**-**	**-**	**-**	**0.013**	
	25a/25b [a,c]	Pair vs 25a	**<0.0001**	**<0.0001**	**<0.0001**	**0.002**	**0.002**	**-**	**0.03**	The pair displayed synergistic virulence phenotype
		Pair vs 25b	**<0.0001**	**<0.0001**	**<0.0001**	**<0.0001**	**<0.0001**	**-**	**0.004**	
	171/172 [a,c]	Pair vs 171	0.65	**0.003**	0.61	-	-	-	0.80	Curve of the pair was different to both of the curves of the single strains (one assay). Unconfirmed by other assays and TD_{50}s comparisons. Absence of synergistic virulence.
		Pair vs 172	0.14	**0.042**	0.89	-	-	-	1.0	
	388c/404c [a,b]	Pair vs 388c	0.70	0.87	0.49	-	-	-	0.49	Similar virulence phenotypes
		Pair vs 404c	0.39	0.25	0.15	-	-	-	0.75	

77c/25b [b]	Pair vs 25b	0.72	0.61	0.15	-	-	-	0.64	Worms with the pair displayed a shorter lifespan than with one single strain only (see supplemental Figure 1). Absence of synergistic virulence phenotype
	Pair vs 77c	**0.005**	**0.006**	0.13	-	-	-	0.64	
25b/26b [b]	Pair vs 25b	0.48	0.76	0.81	-	-	-	0.80	Similar virulence phenotypes
	Pair vs 26b	0.16	0.21	0.35	-	-	-	1.0	
25a/45 [b]	Pair vs 25a	0.10	**0.001**	**0.0004**	-	-	-	0.27	worms with the pair displayed a shorter lifespan than with one single strain only (2 assays out of 3); absence of synergistic virulence (see Supplemental Figure 1)
	Pair vs 45	0.38	0.80	0.58	-	-	-	1.0	
76c/40 [b]	Pair vs 76c	**0.056**	**0.002**	0.11	-	-	-	0.14	worms with the pair displayed a shorter lifespan than with one single strain only (2 assays out of 3); absence of synergistic virulence (see Supplemental Figure 1)
	Pair vs 40	0.16	0.16	**0.02**	-	-	-	0.12	
76c/25b [c]	Pair vs 76c	**<0.0001**	**<0.0001**	**<0.0001**	**-**	**-**	**-**	**0.007**	The pair displayed synergistic virulence phenotype
	Pair vs 25b	**<0.0001**	**<0.0001**	**<0.0001**	**-**	**-**	**-**	**0.04**	
76c/137b [c]	Pair vs 76c	**<0.0001**	**<0.0001**	**<0.0001**	**-**	**-**	**-**	**0.0016**	The pair displayed synergistic virulence phenotype
	Pair vs 137b	**<0.0001**	**<0.0001**	**<0.0001**	**-**	**-**	**-**	**0.005**	
76c/26b [c]	Pair vs 76c	0.14	0.48	**0.001**	-	-	-	0.74	Worms with the pair displayed a shorter lifespan than with one single strain only (1 assay out of 3, see supplemental Figure 1). Absence of synergistic virulence phenotype
	Pair vs 26b	0.11	0.14	0.14	-	-	-	0.90	
76c/44 [c]	Pair vs 76c	0.40	0.48	0.052	-	-	-	0.21	Similar virulence phenotypes
	Pair vs 44	0.16	0.09	00.13	-	-	-	0.38	
76c/404c [c]	Pair vs 76c	**<0.0001**	**<0.0001**	**<0.0001**	**-**	**-**	**-**	**0.014**	The pair displayed synergistic virulence

		Pair vs 404c	**<0.0001**	**<0.0001**	**<0.0001**	**-**	**-**	**-**	**0.032**	phenotype
	76c/388c [c]	Pair vs 76c	0.11	0.45	0.52	-	-	-	0.092	Similar virulence phenotypes
		Pair vs 388c	0.29	0.25	0.11	-	-	-	0.35	
	25a/77c [c]	Pair vs 25a	**<0.0001**	**0.005**	**<0.0001**	**-**	**-**	**-**	**0.007**	The pair displayed synergistic virulence phenotype
		Pair vs 77c	**<0.0001**	**<0.0001**	**<0.0001**	**-**	**-**	**-**	**0.022**	
	25a/137b [c]	Pair vs 25a	0.06	**0.013**	0.10	-	-	-	0.82	Curves of the pair were different to both of the curves of the single strains (1 assay out of 3), but shorter to curves of only one of the single strains (137b). Absence of synergistic virulence.
		Pair vs 137b	**<0.0001**	**<0.0001**	**<0.0001**	-	-	-	0.14	
	76c/25a [c]	Pair vs 76c	0.94	**<0.0001**	**0.0006**	**0.0012**	**<0.0001**	**-**	**0.0008**	Shorter lifespan with the pair compared to both of the curves of the single strains (3 assays out of 5), but unconfirmed by TD$_{50}$s comparisons and 2 other assays.
		Pair vs 25a	0.17	0.087	**0.024**	**0.0038**	**<0.0001**	-	0.12	
	25a/404c [c]	Pair vs 25a	0.33	0.10	0.59	-	-	-	0.82	worms with the pair displayed a shorter lifespan than with one single strain only (2 assays out of 3); absence of synergistic virulence phenotype
		Pair vs 404c	0.13	**<0.0001**	**<0.0001**	-	-	-	0.15	
Inoculum ratio effect on synergy (Figure 4)		Pair (11:1) vs 76c	**0.040**	-	-	-	-	-	-	Shorter survival when the pair was inoculated at ratios of 11:1 or1:100
		Pair (1:100) vs 77c	**0.009**	-	-	-	-	-	-	
		Pair (1:3) vs pair (1:1)	**0.048**	-	-	-	-	-	-	Shorter survival when the pair was at ratio of 1:3

[a] Natural pairs

[b] Pairs composed of strains belonging to the same species

[c] Pairs composed of strains from different species
